# Comparative analysis of targeted long read sequencing approaches for characterization of a plant’s immune receptor repertoire

**DOI:** 10.1186/s12864-017-3936-7

**Published:** 2017-07-26

**Authors:** Michael Giolai, Pirita Paajanen, Walter Verweij, Kamil Witek, Jonathan D. G. Jones, Matthew D. Clark

**Affiliations:** 1 Earlham Institute (EI), Norwich Research Park, Norwich, NR4 7UZ UK; 2 The Sainsbury Laboratory, Norwich Research Park, Norwich, NR4 7UH UK; 3 John Innes Centre, Norwich Research Park, Norwich, NR4 7UH UK; 4 School of Environmental Sciences, University of East Anglia, Norwich Research Park, Norwich, NR4 7TJ UK

**Keywords:** Targeted capture, Gene enrichment, Oxford Nanopore technologies, MinION, PacBio, RenSeq, R-gene, NLR, Resistance gene, Resistance protein, NLR gene fusions

## Abstract

**Background:**

The Oxford Nanopore Technologies MinION™ sequencer is a small, portable, low cost device that is accessible to labs of all sizes and attractive for in-the-field sequencing experiments. Selective breeding of crops has led to a reduction in genetic diversity, and wild relatives are a key source of new genetic resistance to pathogens, usually via NLR immune receptor-encoding genes. Recent studies have demonstrated how crop NLR repertoires can be targeted for sequencing on Illumina or PacBio (RenSeq) and the specific gene conveying pathogen resistance identified.

**Results:**

Sequence yields per MinION run are lower than Illumina, making targeted resequencing an efficient approach. While MinION generates long reads similar to PacBio it doesn’t generate the highly accurate multipass consensus reads, which presents downstream bioinformatics challenges. Here we demonstrate how MinION data can be used for RenSeq achieving similar results to the PacBio and how novel NLR gene fusions can be identified via a Nanopore RenSeq pipeline.

**Conclusion:**

The described library preparation and bioinformatics methods should be applicable to other gene families or any targeted long DNA fragment nanopore sequencing project.

**Electronic supplementary material:**

The online version of this article (doi:10.1186/s12864-017-3936-7) contains supplementary material, which is available to authorized users.

## Background

During pathogen exposure plant cell surface pattern recognition receptors (PRRs) recognize pathogen associated molecular patterns (PAMPs) and trigger a first host immune response termed PAMP- or pattern- triggered immunity (PTI) [1]. Adapted pathogens can weaken PTI with effector molecules that suppress the plant immune response. The plant can detect such effectors via intracellular nucleotide-binding leucine-rich repeat (NLR) proteins, which interfere directly or indirectly with the pathogen effector molecules. This mechanism is called effector-triggered immunity (ETI) and often results in the induction of a cell death response called ‘hypersensitive response’ (HR). Failure of ETI often leads to successful colonization [2–4]. Plant resistance-genes (R-genes) usually encode NLR proteins which are immune receptors that provide the genetic basis of effector-triggered immunity.

R-genes are a valuable resource for plant disease control via breeding: with introduction of resistance alleles by crossing or transgenic strategies crops can be made resistant to pathogens [5]. Plant breeding is time consuming and dependent on the availability of sexually compatible plants containing the desired R-gene sequences. The application of these approaches is limited and pathogen evolution rates may outpace the rate at which resistant plant varieties can be generated [5–7]. An alternative to this is the engineering of transgenic plant varieties [5].

In plant genomes NLR-encoding genes can appear in clusters of multiple genes with nearly identical sequences [8]. Recently three studies [9–11] report on improved approaches for the characterisation and cloning of plant R-genes. Witek et al. cloned resistance genes for potato late blight using RenSeq in combination with PacBio RSII long read sequencing [11]. PacBio RSII based resistance gene enrichment sequencing (RenSeq), termed ‘SMRT RenSeq’ enables the targeted capture of the entire coding sequence of NLR genes and adjacent inter- and intragenic regions which improves the differentiation of similar NLR genes within these clusters. Current SMRT RenSeq protocols using P6-C4 chemistry with movie times of 4 h allow the targeted capture of NLR gene sequences with an insert size of up to 7 kb [12].

PacBio (www.pacb.com) single-molecule real-time (SMRT) sequencing is based on a sequencing by synthesis reaction using a polymerase, generating a mean read length of 10–15 kb for the newest P6-C4 chemistry and approximately 350–500 megabase pairs (Mbp) yield per SMRT-cell. A PacBio library consists of insert molecules with hairpin adapters called SMRT-bells ligated to the each end leading to the formation of a DNA circle, thus the polymerase can sequence the library insert molecule multiple times [13, 14]. For each pass the sequencing information is available as subread (SR) data with approximately 15% error rate. Using multiple sequence passes and a consensus algorithm [14] the read accuracy increases by combining the information of the SRs to a single sequence called read of insert (RoI) which can be over 99% accurate . Advantages of PacBio sequencing are the maturity of the platform including the consistent SMRT-cell yields which are further increased by the newer PacBio Sequel system. Application of PacBio sequencing however requires a specialised laboratory with the necessary capital investment in equipment.

In contrast to the large PacBio RSII or the PacBio Sequel machines, the Oxford Nanopore Technologies (ONT) MinION (www.nanoporetech.com) is a small mobile sequencer powered by a single USB 3.0 port. Sequencing is performed using a disposable flow cell containing an array of nanopores. The sequence information of each strand is acquired separately. Therefore different read types can be distinguished: Lower quality template reads which contain only the sequencing information of the first DNA strand, similar complement reads are composed of the sequencing information of the reverse complement strand and the highest quality 2D reads which are the consensus sequence from both strands of a DNA molecule. MinION sequencing and data analysis are performed in real time. Base-calling is typically performed using a cloud based base-calling algorithm over the Metrichor Desktop Agent software [15], recently local base calling software has become available [16]. During sequencing, the data stream from each nanopore is reported separately for each pore. Reversing of the voltage across a pore can lead to rejection of the DNA molecules, leading to the concept for ONT sequencing termed ‘Read Until’ described in [17] which makes use of these two features for selective sequencing - briefly: real-time squiggle data is continuously compared to a user provided reference containing simulated squiggle data leading to ejection of the DNA strand from the pore by reversing the voltage [17]. To date this has only been demonstrated for a small reference data set, but with improvements one could envisage experiments that reject reads not containing NLR gene motifs, or already known NLR gene sequences. This unique feature would remove the need of capture bait design and specialized library preparation from most targeted sequencing approach e.g. RenSeq, Cancer gene panels, common human pathogens, genotyping, exome resequencing etc.

The MinION manufacturer’s genomic DNA sequencing protocols are optimised for an insert size of 8 kb (using MAP-SQK006 reagents). Recently average 2D read lengths of 10 kb with sequence reads up to 58 kb were reported [18]. With its small size, low cost, and long reads the ONT MinION enables immediate in situ analyses removing the need for sample shipping and preservation. This makes the MinION an attractive alternative to the PacBio – especially when sequencing of small genomes or targeted enrichment sequencing strategies are of interest. MinION’s long read length also enables the resolving of complex genomic regions e.g. NLR gene clusters. As SMRT RenSeq is finding increasing attention and application in plant R-gene cloning we compared the performance of the MinION to the PacBio RSII in RenSeq experiments. The results are likely to also be useful for similar hybridisation enrichment procedures e.g. other gene families, exome sequencing.

We reproduced the SMRT RenSeq experiment by Witek et al. [11] using Nanopore sequencing (ONT MinION R7.3 chemistry), tested assembly methods and compared the MinION results to the reported PacBio RSII dataset. Finally using an in silico experiment we show that the MinION is able to identify novel NLR genes from a sample.

## Results

### Sequencing

**Fig. 1:**
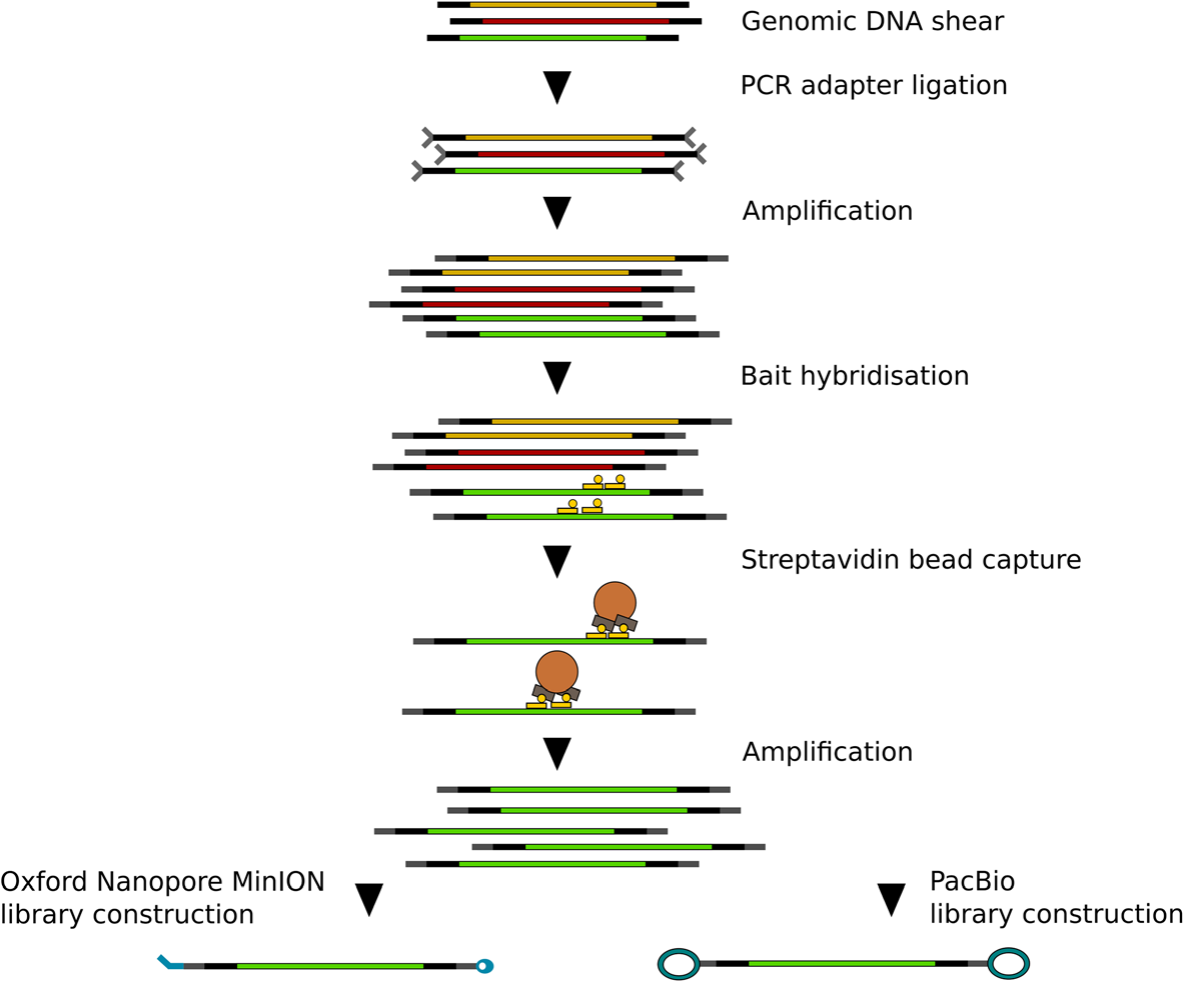
Overview of the RenSeq protocol: Genomic DNA is sheared to the desired insert size. PCR adapters are ligated to the genomic DNA shear and the shear is amplified. Biotinylated custom made baits are hybridised to the sequences of interest. Molecules with hybridised baits can be separated by Streptavidin magnetic bead capture. The captured DNA sequences are subsequently amplified and the amplified products processed to a MinION library or a PacBio library

To create a comparable MinION dataset to the published SMRT RenSeq [11] (Fig. 1) we amplified the same captured *Solanum americanum* DNA (this has Illumina adapters and has been amplified once, but is before PacBio adaptor addition). Using three PCR reactions we obtained 10.6 μg DNA with a modal library size of 2.9 kb after AMPure XP bead DNA cleanup (Additional file 1: Figure S1).

We constructed two sequencing libraries, each library is sufficient for two flow cells, and we generated data from four R7.3 flow cells with 48 h sequencing runs to yield a total of 193,850 Template (503 Mbp), 193,850 Complement (484 Mbp) and 193,850 2D reads (503 Mbp) passing filter (Table 1). In further analysis this data is compared with P6-C4 chemistry PacBio read data (383,981 PacBio SR and 101,331 RoI) sequenced on three individual SMRT cells from this same sample, as described in [11].

**Table 1:** Comparison of ONT MinION R7.3 and PacBio RSII sequencing performance values: MinION fail and pass and PacBio RSII SR and RoI amount of reads, read quality and read size

	MinION fail Template	MinION fail Complement	MinION fail 2D	MinION pass Template	MinION pass Complement	MinION pass 2D	PacBio Subreads	PacBio Reads of Insert
Number of reads [n]	268,044	112,405	83,692	193,850	193,850	193,850	383,981	101,331
Number of bases [Mbp]	630	273	209	507	484	503	1360	353
Modal accuracy	74.88%	60.19%	84.15%	74.88%	74.88%	92.06%	90.00%	99.99%
Mean accuracy	70.24%	66.42%	82.62%	77.84%	76.79%	91.36%	89.83%	99.57%
N50 reads length [bp]	2916	2829	2786	2278	2169	2262	3540	3559
Mean read length [bp]	11,665	10,121	3250	2838	2716	2813	3818	3675
Modal read length [bp]	1138	2454	2306	2570	2720	2586	3482	3485

### Read quality comparison

**Fig. 2:**
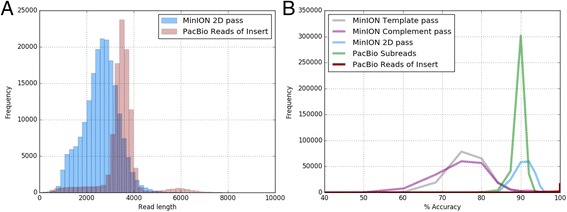
Performance comparison between ONT MinION and Pacbio RSII: **a** Read length profile of MinION 2D pass reads obtained on four R7.3 flow cells with a 3 kb PCR product and PacBio RoI obtained by Witek et al. [11]. **b** Accuracy scores of MinION pass reads and PacBio SR and RoI (the PacBio RoI mostly possessing an accuracy of 99% are visible as read peak at the 100% mark)

We assessed the quality of the failed and passed MinION reads (template, complement and 2D) as well as PacBio SR and RoI. Our MinION 2D pass reads had a modal accuracy of 92.06% and mean accuracy of 91.36% which is more similar to the quality of PacBio subreads (modal SR accuracy: 90.00%, mean SR accuracy: 89.89%) than PacBio RoI: modal accuracy of 99.99% and mean accuracy of 99.57%. (Table 1, Fig. 2). We also observed a shorter insert size from the MinION data i.e. 2.8 kb for 2D pass reads (lower for the other read types) (Table 1, Fig. 2) in comparison to the PacBio data (3.5 kb insert size) (Table 1, Fig. 2). As the MinION 2D pass reads are the most accurate MinION sequence type, and PacBio RoI the most accurate PacBio sequence type, we base the following analysis exclusively on 2D pass reads and PacBio RoI.

### Read processing and assembly

**Fig. 3:**
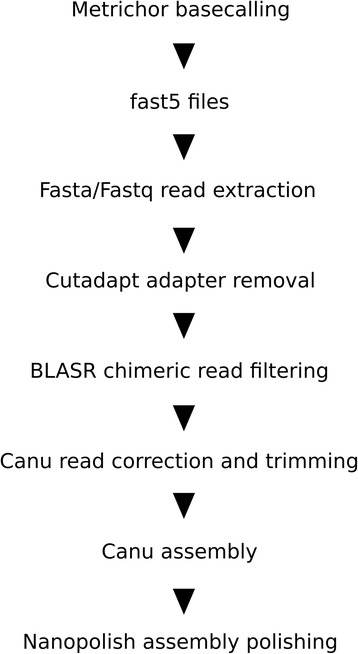
MinION 2D read assembly pipeline: Basecalling is performed using Metrichor. The FASTA or FASTQ information is extracted from the fast5 files. PCR adapters are removed using cutadapt and chimeric reads are filtered out of the dataset using BLASR. The adapter curated reads are corrected and trimmed in the Canu assembly pipeline and assembled with Canu. After assembly the contigs are polished with nanopolish

As a consequence of the library preparation protocol each sequence carries Illumina adapters on the 5′ and 3′ ends. These adapter sequences allow the amplification of the library, but can interfere with the assembly. Further, PCR induced fusions can lead to chimeric molecules which are connected by an adapter sequence. To avoid interference of the Illumina adapter sequences with the assembly process and chimeric molecules we remove adapters prior to sequence assembly. As the quality of the MinION 2D pass reads was lower than the quality of the PacBio RoI data, we applied read correction of adapter curated molecules and a final contig polishing step of the Oxford Nanopore data assembly. Our pipeline is therefore composed of the following steps: adapter trimming, chimeric read filtering, long read correction, long read assembly and contig polishing steps (Fig. 3).

Trimming and chimeric read filtering reduced the number of MinION 2D pass reads from 193,850 to 193,724 reads and the number of PacBio RoI from 101,331 to 100,958 (Table 2). The published PacBio RSII dataset was assembled using the commercial software Geneious and hand-curated with Illumina MiSeq 250 bp PE data [11]. For the assembly of lower accuracy MinION sequencing data we used Canu, which is based on the Celera Assembler [19] and adapted for long and less accurate reads [20, 21]. We also assembled the adapter trimmed PacBio RoI with Canu. The Canu pipeline is based on three different steps: (1) Detection of overlaps in low-accuracy sequences and generation of a corrected sequence consensus, (2) quality trimming of the corrected reads, (3) assembly of the trimmed sequences [20].

**Table 2:** Read statistics after adapter trimming, chimera filtering and correction: Adapter trimming, removal of reads smaller than 150 bp and chimera filtering reduced the number of Mbp in each dataset by approximately 5%. Due to the lower quality MinION reads were Canu corrected before assembly. The Canu pipeline further reduced the amont of MinION reads to 304 Mbp before assembly – a number similar to the amount of PacBio RoI

	MinION 2D pass (trimmed, filtered)	MinION 2D pass (trimmed, filtered, corrected)	PacBio Reads of Insert (trimmed, filtered)
Number of reads [n]	193,724	114,027	100,958
Number of bases [Mbp]	475	304	337
N50 reads length [bp]	2681	2739	3430
Mean read length [bp]	2681	2784	3536

To assemble the MinION 2D pass reads we used the Canu correction and quality trimming function (hereafter corrected MinION 2D pass reads), for the PacBio RoI we did not include this step due to the high sequence accuracy of RoI reads. After Canu correction and trimming of the MinION 2D pass reads we retained 114,027 2D reads, 304 Mb of data, which compares well to 100,958 PacBio RSII RoI (337 Mb data) (Table 2).

**Fig. 4:**
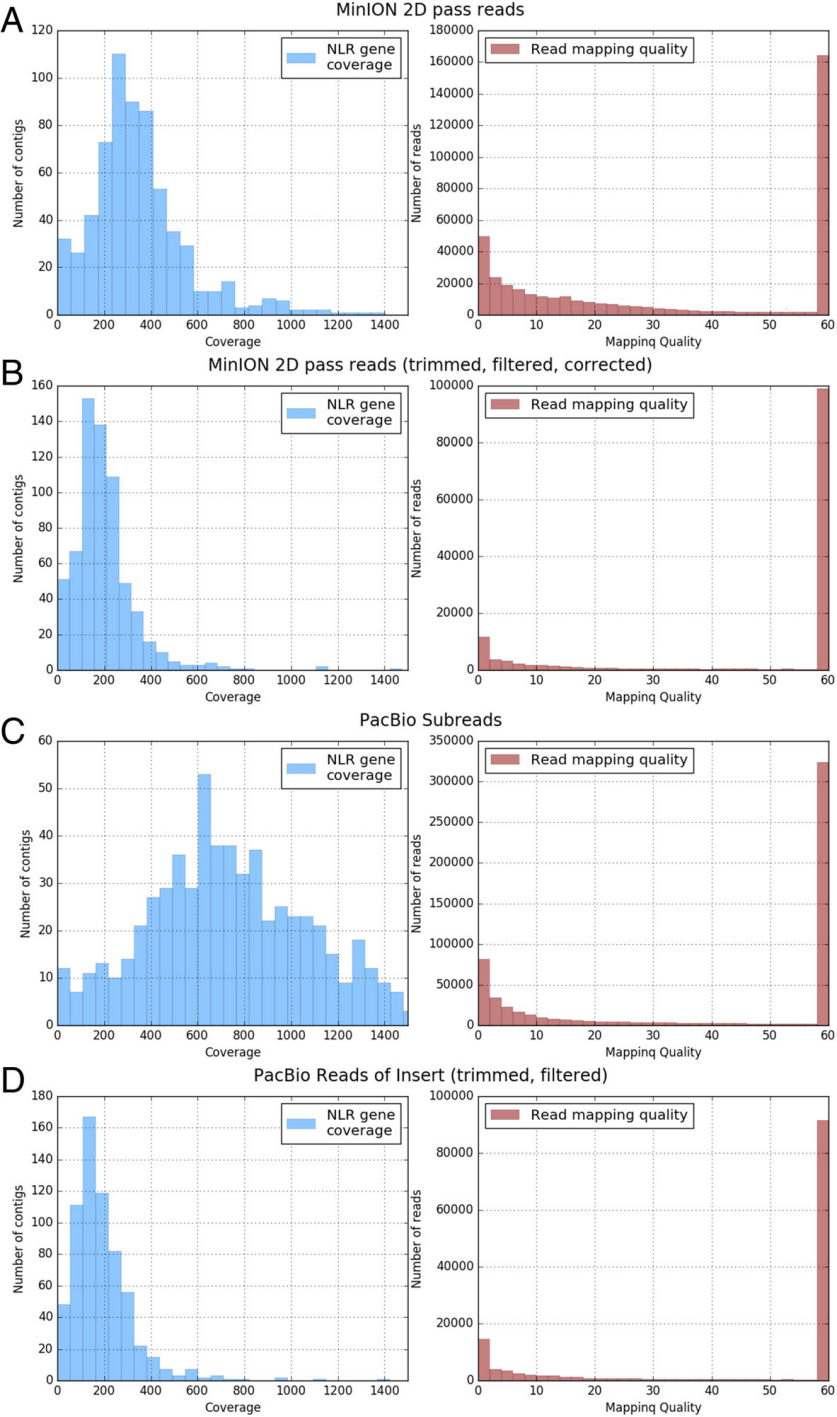
Coverage (blue) and mapping quality (red) histograms of MinION and PacBio reads mapped to the 649 annotated NLR genes: **a** MinION 2D pass reads **b** Adapter trimmed, chimera filtered, corrected MinION 2D pass reads, **c** PacBio SR, **d** Adapter trimmed, chimera filtered, PacBio RoI. Approximately 40 contigs are covered with <50× by the MinION 2D and PacBio RoI datasets. For most of the contigs the coverage is ≥50×. For the coverage histograms a cutoff at 1500× was defined. Whereas all datasets are containing some low quality mapping reads indicating ambiguous mapping due to the high similarity of NLR genes, the majority of reads is mapping with a Phred score of 60. As expected PacBio SR and MinION 2D pass reads show a higher number of low quality mapping events as adapter trimmed and chimera filtered PacBio RoI and adapter trimmed, chimera filtered and corrected MinION 2D pass reads

Witek et al. annotated and manually corrected 649 NLR gene contigs of *S. americanum* accession SP2271 [11]. We used these curated sequences as our reference dataset. To analyse the quality of the uncorrected and corrected MinION 2D pass reads, as well as PacBio SR and RoI reads we mapped the reads to the annotated 649 complete NLR-encoding genes. Of the corrected MinION 2D pass and PacBio RoI datasets more than 95% of the reads mapped to the full length NLR genes leading to a mean coverage of 55.94 for the corrected MinION 2D pass reads and 55.48 for the PacBio RoI data. The coverage frequency histograms (Fig. 4) show a coverage of at least 50 x for the majority of the 649 NLR genes with either MinION 2D pass reads or PacBio RoI. However, a small number of contigs (approximately 40) were covered with less than 50 x. Error rates for the mapped but uncorrected MinION 2D pass reads and lower quality PacBio SR are 12.96% and 13.45% respectively. Error rates of mapped reads decreased to 3.73% for the corrected MinION 2D pass reads, very close to the rate of 3.93% for PacBio RoI, suggesting that our MinION correction pipeline works well (Table 3). Plotting of the mapping quality scores indicated a high mapping quality score (60) for most of the reads, with lower quality mapping reads for uncorrected MinION 2D pass reads and PacBio SR. (Fig. 4).

**Table 3:** Mapping statistics of reads before and after filtering to the 649 annotated NLR genes: Not adapter filtered MinION 2D pass and PacBio SR and adapter filtered MinION 2D pass (Canu corrected and trimmed) reads and PacBio RoI were mapped to the annotated 649 NLR genes described by Witek et al.

	MinION 2D pass	MinION 2D pass (trimmed, filtered, corrected)	PacBio Subreads	PacBio Reads of Insert (trimmed, filtered)
Mapped reads	99.13%	97.27%	97.38%	95.54%
Mean Coverage	82.32	55.94	210.80	55.48
Mean mapping quality	45.53	50.50	47.63	52.11
General error rate	13.49%	3.73%	13.45%	3.93%

To determine on-target capture rates we counted the number of reads containing a bait sequence over 96 bases with at least 80% accuracy as described by Jupe et al. [22]. We assessed the sequence similarity between reads and baits using BLAST [23]. 81.98% of the corrected MinION 2D pass and 73.73% of the PacBio RoI contained bait sequences. For PacBio SR and MinION 2D pass reads these numbers were lower (62.50% for uncorrected MinION 2D pass and 57.82% for PacBio SR) presumably due to the effect of higher read error rates (Table 4).

**Table 4:** Comparison of read statistics before and after adapter curation: NLR-Parser statistics of not adapter filtered MinION 2D and PacBio SR and adapter filtered MinION 2D pass (Canu corrected and trimmed) reads and PacBio RoI

	MinION 2D pass	MinION 2D pass (trimmed, filtered, corrected)	PacBio Subreads	PacBio Reads of Insert (trimmed, filtered)
Number of reads containing baits	121,170	93,482	219,934	74,442
% of reads containing baits	62.50%	81.98%	57.28%	73.73%
NLR-Parser hits	20,525	56,211	11,003	45,853
% NLR-Parser hits of total reads	10.59%	49.30%	2.86%	45.41%
NLR-Parser hits scored as partial	19,984	50,410	10,791	39,512
% NLR-Parser hits scored as partial	97.36%	89.68%	98.07%	86.17%
NLR-Parser hits scored as complete	541	5801	212	6341
% NLR-Parser hits scored as complete	2.64%	10.32%	1.93%	13.83%

To assess the percentage of NLR genes in the read datasets we used the NLR-Parser [24] software to predict NLR genes based on their motifs. As the software is sensitive to indels which cause frameshifts we used only corrected MinION 2D pass reads (49.30% sequences contained NLR protein motifs of which 10.32% were complete), and the PacBio RoI data (45.41% contained NLR motifs of which 13.83% were listed as complete). The higher number of complete annotated reads in the PacBio RoI data is consistent with the longer sequenced insert sizes (Table 4).

We assembled the corrected MinION 2D pass and the PacBio RoI with Canu. After assembly of the MinION 2D pass reads the contigs were further corrected with nanopolish [25]. We also performed PacBio data HGAP assembly [26] for RoI data using a pipeline which has been modified for PacBio RenSeq data [12]. We obtained 1085 contigs (7.74 Mb) for the nanopolished MinION 2D pass Canu assembly, 1483 contigs (9.14 Mb) for the PacBio RoI Canu assembly, 1460 contigs (8.31 Mb) for the PacBio HGAP assembly and 837 contigs (9.01 Mb) for the PacBio Geneious assembly (Table 5). The N50 values of the Canu and HGAP assemblies were both shorter than the 10,935 bp N50 length of the Geneious assembly: 4958 bp nanopolished Canu MinION 2D pass, 4464 bp Canu PacBio RoI, 3949 bp HGAP PacBio RoI. The average contig size of the Canu assemblies is comparable with the Geneious assembly size of 13,929 bp: 12,366 bp nanopolished Canu MinION 2D pass, 10,099 bp Canu PacBio RoI, 9353 bp HGAP.

**Table 5:** Assembly statistics and NLR-Parser evaluation of the assemblies: Canu assembly (nanopolished and not nanopolished) using MinION 2D pass data, Canu using PacBio RoI, HGAP using PacBio RoI and Geneious PacBio RoI

	Canu MinION	Canu MinION (nanopolish)	Canu PacBio	HGAP	Geneious
Number of contigs	1085	1085	1483	1460	837
Minimal contig length [bp]	1568	1695	1008	517	3882
Contig N80 [bp]	4873	4958	4464	3949	7775
Contig N50 [bp]	8089	8230	6817	7149	10,935
Contig N20 [bp]	13,963	14,185	12,785	11,796	18,321
Mean contig size	12,167	12,366	10,099	9353	13,929
Maximal contig length (bp)	132,431	134,631	59,085	85,187	55,450
Sum of bp assembled	7,606,604	7,749,213	9,835,757	8,307,997	9,008,910
NLR-Parser hits	584	608	557	667	586
NLR-Parser hits scored as partial	308	332	324	372	257
% NLR-Parser hits scored as partial	52.74%	54.60%	58.35%	55.69%	43.78%
NLR-Parser hits scored as complete	275	276	231	295	329
% NLR-Parser hits scored as complete	47.26%	45.39%	41.65%	44.31%	56.21%

**Fig. 5:**
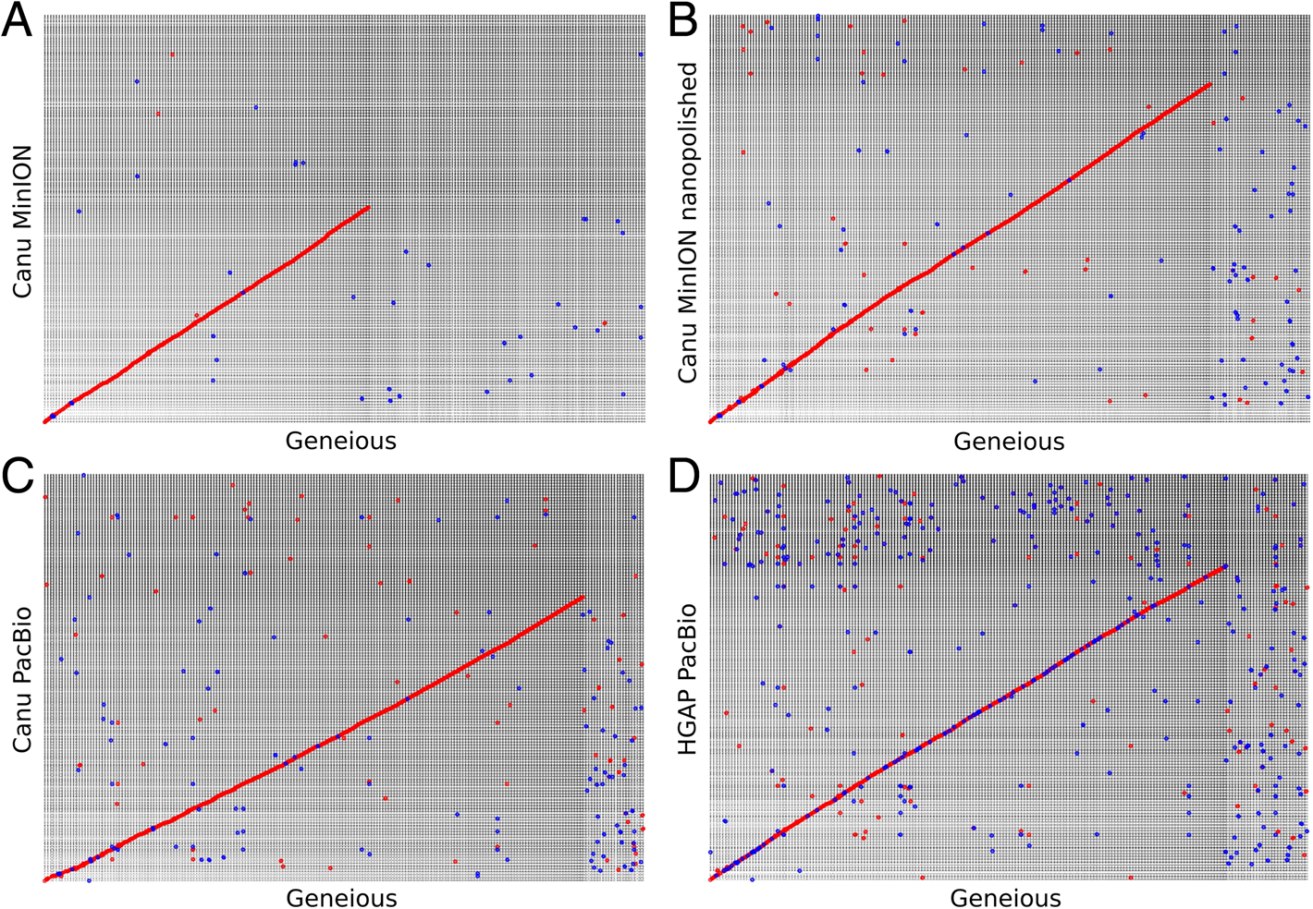
NUCmer comparison of assemblies vs. Geneious: All assemblies were aligned to the Geneious reference assembly using NUCmer and visualized using mummerplot. **a** Canu MinION 2D pass assembly, **b** Nanopolished Canu MinION 2D pass assembly, **c** Canu PacBio assembly, (**D**) HGAP assembly. A remarkable increase in identity was achieved by nanopolishing the Canu MinION assembly visible in (**a**). Red dots indicate forward matches, blue dots indicate reverse matches. Contig names on the x and y axis were removed as due to the high number of contigs the names were not resolved properly

We aligned all our assemblies to the Geneious reference using NUCmer (minimum length of a single match was set to 500) and visualised the alignments using mummerplot [27]. Of all assemblies the nanopolished Canu MinION 2D pass assembly most closely resembled the manually corrected Geneious reference. A remarkable increase of identity between the Canu MinION assemblies and the reference was achieved by nanopolishing the data (Fig. 5).

### Assembled NLR-encoding gene content evaluation

To count the number of NLR-encoding contigs we analysed the assemblies with the NLR-Parser [24]. For the Canu MinION 2D pass assembly without nanopolishing we obtained 584 total NLR-Parser hits of which 275 are described as complete. After nanopolishing this increased to 608 NLR-Parser hits of which 276 are complete, indicating that the quality increase from sequence polishing leads to the recognition of more motifs by the MAST [28] based NLR-Parser software. The Canu PacBio assembly contained 557 contigs with 231 complete hits. Only the HGAP assembly outscored the nanopolished Canu MinION assembly with 667 NLR motif encoding contigs (295 complete). The Geneious assembly contained 586 contigs with NLR motifs of which 329 were scored as complete (Table 5).

To assess if the assemblies produced the same NLR gene repertoire and to determine the length and percent identity in comparison to the annotated NLR dataset we used BLAST. We searched the assemblies using the 649 published and annotated NLR encoding sequences and evaluated only the best BLAST hit with the criteria e-value = 0. We manually inspected the BLAST results to avoid NLR query sequences aligning to the same region in a contig and generating falsely positives. In the Geneious reference dataset the annotated NLRs share 100.00% ± 0.00% identity on an average alignment length of 7811 bp ± 2166 bp (99.98% ± 0.49% query alignment length percentage) and result in 649 BLAST hits. For the PacBio data HGAP assembly this results were 99.85% ± 0.70% identity over 6729 bp ± 1742 bp (86.53% ± 14.54% query alignment length percentage) and 606 BLAST hits (93.97% Geneious database coverage). The Canu PacBio assembly possessed 99.62% ± 1.21% identity over 6501 bp ± 2000 bp (83.26% ± 19.70% query alignment length percentage) and 577 BLAST hits (88.90% Geneious database coverage). The nanopolished Canu MinION assembly produced 99.42% ± 0.50% identity over 6989 bp ± 1700 bp (89.28% ± 13.28% query alignment length percentage) and 594 BLAST hits (91.52% Geneious database coverage). The non-nanopolished Canu MinION 2D pass dataset in contrast scored 98.06% ± 0.61% identity over 6943 bp ± 1718 bp (88.93% ± 13.45% query alignment length percentage) with 591 BLAST hits (91.06% Geneious database coverage) indicating that nanopolishing draft Canu assemblies of MinION 2D pass data increases the assembly quality by an average of 1.37% (Table 6) similar to results reported in [21].

**Table 6:** Comparison of all assemblies with the annotated NLR genes using BLAST: The 649 NLR genes described by Witek et al. were mapped to each assembly. In all cases all 649 NLR genes are mapping in the assemblies

	Canu MinION	Canu MinION (nanopolish)	Canu PacBio	HGAP	Geneious
Average percent identity	98.61% ± 0.61%	99.42% ± 0.50%	99.62% ± 1.21%	99.85% ± 0.70%	100.00% ± 0.00%
Average alignment length	6943 bp ± 1718 bp	6989 bp ± 1700 bp	6501 bp ± 2000 bp	6729 bp ± 1742 bp	7811 bp ± 2166 bp
BLAST hits	591	594	577	606	649
% database covered	91.06%	91.52%	88.91%	93.37%	100%

We also analysed the assemblies for encoded NLR proteins using AUGUSTUS [29] to de novo predict protein sequences and then NLR-Parser to identify NLR proteins [24]. We identified 649 NLR proteins (475 partial / 174 complete) for the nanopolished Canu MinION data, 611 NLR proteins (361 partial / 251 complete) for the Canu PacBio data, 805 NLR proteins (495 partial, 310 complete) for the HGAP data and 702 NLR proteins (380 partial, 322 complete) for the Geneious data. We searched the protein sequences of the nanopolished Canu MinION, Canu PacBio, Geneious and HGAP derived data with the predicted proteins of the 649 NLR reference (641 NLR proteins, 325 partial, 316 complete) using BLASTP and hand-curating the BLASTP results to exclude false positive produced by query sequences aligning to the same subject sequence. This resulted in 368 BLASTP hits with an average percent identity of 90.67% ± 9.06% and an average alignment length of 826 amino acids ±278 amino acids (84.11% ± 18.18% query alignment length percentage) for the nanopolished Canu MinION assembly. For the Canu PacBio assembly we counted 445 hits with 98.19% ± 5.59% average identity and 914 amino acids ±296 amino acids alignment length (93.81% ± 13.62% query alignment length percentage). The numbers increased for the HGAP assembly to 533 hits with 99.22% ± 2.98% average identity and 954 amino acids ±299 amino acids (95.50% ± 10.82% query alignment length percentage) alignment length (Table 7). The Geneious data produced 587 BLASTP hits with 99.87% ± 1.12% average identity and 971 amino acids ±330 amino acids alignment length (98.34% ± 6.12% query alignment length percentage) (Table 7).

**Table 7:** AUGUSTUS protein prediction results: BLASTP comparison of R-proteins predicted of all assemblies with the 641 predicted R-proteins of the 649 NLR gene reference (amino acids is abbreviated with aa)

	Canu MinION (nanopolish)	Canu MinION (nanopolish, pilon)	Canu PacBio	HGAP	Geneious
Predicted R-proteins	649	675	611	805	702
NLR-Parser complete	174	283	251	310	380
NLR-Parser partial	475	392	361	495	322
R-proteins with BLASTP hit	368	496	445	533	587
% Database coverage	57.41%	77.37%	69.42%	83.15%	91.57%
% identity to reference	90.67% ± 9.06%	95.53% ± 8.42%	98.19% ± 5.59%	99.22% ± 2.98%	99.87% ± 1.12%
BLASTP alignment length of R-proteins	826aa ± 278aa	921aa ± 301aa	914aa ± 296aa	954 aa ±299 aa	971 aa ±330 aa
BLASTP % alignment length of R-proteins	84.11% ± 18.18%	93.65% ± 13.74%	93.81% ± 13.62%	95.50% ± 10.82%	98.30% ± 6.12%

This result indicates that assemblies produced with highly accurate PacBio RoI data performed better in the protein prediction analysis than the Canu MinION assembly. Further we see a higher similarity between the predicted proteins of the HGAP PacBio assembly and the 649 NLR reference as for the Canu PacBio assembly.

Due to the similar performance of all assemblies at the nucleotide but not protein level we assumed that the results obtained for the predicted proteins on the nanopolished Canu MinION NLRs are caused by errors in the contigs leading to frameshifts or mispredictions. The errors in the assembled Canu MinION contigs likely stem from the indel rich MinION sequencing error profile. Frameshift mutations caused by indels can lead to false or no de novo protein prediction likely contributing to the difference between the PacBio and MinION data assemblies. An improvement of assemblies can be achieved by contig correction with high-quality reads such as shorter Illumina sequences which are low in indel. To see if contig correction with Illumina read data leads to a higher similarity between the predicted proteins of the 649 NLR reference and the nanopolished Canu MinION datasets we used Pilon [30]. Pilon is described to fix small and large indels, misassemblies and perform gap filling [21, 30, 31]. We counted 22,500 Pilon repair events of which 20,618 (91.63%) were single base insertions, deletions or single base changes. We compared the predicted proteins of the Pilon repaired, nanopolished Canu MinION dataset with the proteins of the 649 NLR reference. We counted 675 NLR proteins (392 partial / 283 complete) which produced 496 hits with 95.53% ± 8.42% average identity and 921 amino acids ±301 amino acids (93.65% ± 13.74% query alignment length percentage) alignment length (Table 7). On the nucleotide level the Pilon repaired data produced similar numbers as the non-Pilon repaired MinION dataset with 616 NLR motif containing contigs (323 partial / 293 complete) being highlighted by the NLR-parser with 99.39% ± 0.48% identity over 6944 bp ± 1724 bp (88.69% ± 13.80% query alignment length percentage) and 598 BLAST hits (92.14% Geneious database coverage) in comparison to the 649 NLR nucleotide reference. Taken together we find that increased MinION data quality e.g. contig polishing with Illumina data, is beneficial in predicting protein coding genes.

### Rapid identification of novel NLR genes

With its rapid library preparation protocol, real time sequencing mode and the capability to sequence large DNA fragments with currently increasing accuracy the MinION is an attractive tool to quickly characterise samples of interest. This could enable rapid generation of high accuracy datasets without the need of a specialised laboratory environment, or expensive sequencing platforms as the PacBio RSII. This could be useful when surveying for areas rich in novel resistances, bypass sample shipment and phytosanitary restrictions by generating sequencing data in situ which could be easily transferred electronically for further analysis, or even creation of synthetic DNA constructs for *in planta* testing.

To date NLR gene identification depends on assembling an entire RenSeq dataset, characterizing the assembly with programs such as the NLR-Parser and manual annotation of the generated contigs [11, 24]. We proposed a rapid strategy for the identification of new NLR genes in a novel dataset i.e. a field experiment, and reasoned this could be achieved by comparing MinION 2D reads as they are generated to a reference NLR gene database. High quality reads that do not align or align with a bad quality to this database potentially contain new gene sequences and these reads should assemble using our pipeline to accurate representations of novel NLR genes and allow quick characterisation of the novel NLR gene repertoire (Additional file 1: Figure S2).

For in the field analysis e.g. using a laptop, we chose lightweight tools such as the NLR-Parser and BLAST. As the raw MinION 2D pass read accuracy is too low for accurate characterization of NLR genes we performed this analysis on the Canu corrected 2D read data. To compare the MinION results with the PacBio data we also performed the analysis on PacBio RoI sequences.

To test our strategy in an in silico experiment we aimed to re-assemble the fusion domain NLR genes described by Witek et al. [11]. We removed the 37 fusion domain containing sequences from the 649 annotated reference NLR genes. We searched this new reference dataset with the Canu corrected 2D reads and the PacBio RoI using BLASTN. To define an empirical threshold for low quality mapping reads we extracted all reads which mapped with a percent identity below two standard deviations from the average mapping percent identity. We assembled these low-quality mapping and all non-mapping reads (MinION: 13,282 reads totalling 32.89 Mb, PacBio: 9077 reads totalling 28.63 Mb). After assembly we filtered the assembled contigs using the NLR-Parser. To reduce time and compute needs we did not perform a nanopolishing step. This resulted in 76 contigs (54 partial, 22 complete) for the Canu corrected 2D reads and 24 contigs (17 partial, 7 complete) for the PacBio RoI. We searched the 649 NLR gene reference database with the obtained contigs using BLAST. 74 MinION and 19 PacBio contigs mapped to the full NLR gene reference dataset. We manually inspected alignments of contigs to fusion domain NLR gene sequences with an e-value of 0. For the MinION dataset we recovered 29 of 37 fusion domain NLR genes with an average percent identity of 98.00% ± 0.91% and an average alignment length of 6060 bp ± 1661 bp resulting in an alignment length percentage of 91.84% ± 14.89% of the assembled contig aligning to the fusion domain NLR genes. For the PacBio dataset we recovered 16 of 37 fusion domain NLR genes with an average percent identity of 99.57% ± 0.47% and an alignment length of 5569 bp ± 1272 bp (alignment length percentage to the reference: 93.07% ± 13.81%). The difference of assembled fusion NLR genes between the MinION and PacBio dataset can stem from the higher number of MinION reads entering the assembly pipeline. In other words, PacBio RoI reads, due to their higher quality, can align with a high score to related sequences and therefore are not considered for assembly, whereas a higher number of MinION reads will enter assembly as more reads (with lower quality and BLAST scores), are not filtered out. This indicates that for this type of analysis, and despite the high sequence similarity of NLR genes [8] the MinION data performs well.

Taken together this suggests that the identification of novel NLR genes using fast and efficient tools such as BLASTN and the NLR-Parser is possible. With R7.3 MinION accuracy we found read correction to be necessary, and developed a pipeline to do so. A similar analysis based on the uncorrected MinION 2D pass reads is not possible due to the low NLR-Parser motif recognition efficiency on uncorrected reads (Table 4). While PacBio systems already provide high accuracy (via RoI sequences), the MinION is currently under intense technological development to provide higher yields and higher accuracy.

## Discussion

With the ONT MinION sequencing device an attractive alternative to PacBio sequencing for generating long reads is emerging. This makes the MinION an interesting tool for repeat rich genomes, genomic regions containing clusters of similar genes e.g. NLR genes. As shown by Witek et al. [11] SMRT RenSeq using the PacBio RSII sequencer is changing the speed of R-gene cloning. Here we show that the MinION can compete with the PacBio RSII in sequencing long insert DNA and found that the accuracy of MinION 2D pass reads on R7.3 flow cells is comparable with PacBio SR. Especially MinION 2D pass reads where both strands of a DNA molecule are sequenced and combined to a single consensus read represent an improvement of sequence accuracy in comparison to the MinION Template and Complement reads which contain the sequencing information of only the first strand or the first and partially the second strand respectively. To assemble NLR genes to over 99% accuracy we therefore propose an assembly strategy of MinION 2D pass reads which is based on read adapter curation using cutadapt [32], chimeric read filtering using BLASR [33], Canu read correction and trimming [20] and further polishing of the assembled contigs using nanopolish [25]. In our study this results in an average MinION contig accuracy of 99.41% in comparison to the manually annotated, PacBio generated reference dataset. We observed a lower percent identity (90.67%) between the predicted proteins of the Canu MinION assembled NLR genes and the predicted proteins of the SP2271 NLR gene reference. We assume that this is caused by contigs containing errors which are leading to protein misprediction.

With the MinION currently under intense technological development, with new pores, chemistries and software, the increasing flow cell yields and an increased sequencing accuracy of newer chemistries, this alltogether will also contribute to better results. New high throughput Nanopore platforms using the same chemistry e.g. GridION X5 and PromethION will enable this analysis on a larger scale, whereas the smartphone run SmidgION will increase portability.

## Conclusion

Although we observed a lower sequencing yield on an ONT R7.3 flow cell in comparison to a PacBio SMRT-cell (117.7 Mbp 2D pass reads and 453.3 Mbp filtered SR respectively), the MinION read length and the Canu MinION 2D pass read assembly accuracy indicate the usefulness of the ONT MinION for studying genomic regions of interest using targeted sequencing. This approach will allow the interrogation of complex genomic regions ranging from the prokaryotic kingdom [34] to eukaryote organisms such as plants [11] and humans [35] - especially with improved ONT sequencing technologies in the near future. Development of software applications such as ‘Read Until’ may further advance ONT platforms to unique sequencing devices for targeted sequencing with the enrichment based on tunable bioinformatics [17].

## Methods

### Targeted capture amplification

We used the same Dynabeads MyOne Streptavidin C1 beads (Thermo Fisher, Cambridge, UK) with the captured DNA fragments as in the experiment described by Witek et al. [11]. We amplified the target sequences with 3 PCR reactions using the following reaction set-up: 1.0 μl Dynabeads MyOne Streptavidin C1 beads, 1.5 μl 10 μM HPLC purified RAD_F primer (5′-AATGATACGGCGACCACCGA-3′) (IDT, Integrated DNA Technologies, Leuven, BE), 10 μl 5× Kapa HiFi Fidelity Buffer, 1.0 μl Kapa HiFi Polymerase (1 U/μl) in a total volume of 50 μl. PCR cycling was performed according to the program: 94 °C 3 min, [94 °C 30 s, 60 °C 30 s, 68 °C 4 min] 25 x, 68 °C 10 min. The ramp rate was set to 3.0 °C/s, the PCR reaction was conducted in a G-Storm GS1 (G-Storm, Somerton, UK) thermal cycler. After amplification the reactions were pooled and cleaned up using a 0.4× AMPure XP bead ratio (Beckman Coulter, Brea, CA, USA) and eluted in 150 μl 1× TE buffer (10 mM Tris-HCl pH 8.0, 1 mM EDTA). The concentration after cleanup was measured on a Qubit2.0 Fluorometer (Thermo Fisher, Cambridge, UK) and indicated a total yield of 10.6 μg. The profile of the amplification peak was assessed on the Agilent 2200 Tapestation (Agilent, Stockport, UK) (Additional file 1: Figure S1). The sample was stored on −20 °C until being processed into MinION sequencing libraries.

### MinION sequencing library construction

Two sequencing libraries were constructed using the MAP-SQK006 reagents kit. End repair was performed using the NEBNext End Repair Module (E6050, NEB, Hitchin, Hertfordshire, UK) by mixing 1.0 μg DNA dissolved in 10 μl 1× TE buffer, 5 μl Quality Control DNA CS, 10 μl 10× NEBNext End Repair Reaction buffer and 5 μl NEBNext End Repair Enzyme Mix in a total reaction volume of 100 μl. The reaction was incubated for 20 min at 24 °C in a G-Storm GS1 (G-Storm) thermal cycler without heated lid. After incubation the reaction was cleaned up using a 0.45× AMPure XP ratio (45 μl AMPure XP beads) and eluted in 26 μl water. 1 μl of each library was used to measure the concentration of the eluate on a Qubit2.0 Fluorometer indicating 750 ng and 630 ng DNA post elution. The DNA was A-tailed using the NEBNext dA Tailing Module (E6053, NEB) by mixing 25 μl eluted DNA with 2 μl Klenow Fragment (3′-5′ exo^−^) and 3 μl 10× NEBNext dA-Tailing Reaction Buffer. The reaction was incubated for 10 min at 37 °C in a G-Storm GS1 thermal cycler without heated lid. After A-tailing we proceeded directly with adapter ligation. The measured DNA concentrations before A-tailing corresponded to 0.39 pmol and 0.33 pmol DNA with a size of 2900 bp. As the SQK-MAP006 protocol suggests to use 0.20 pmol DNA in adapter ligation if insert sizes are below 3 kb, we increased the volumes of Adapter Mix and HP Adapter in the final ligation to proceed with a higher amount of DNA in library preparation. The ligation was therefore performed using 30 μl A-tailed DNA, 4 μl HP Adapter, 16 μl Adapter Mix and 50 μl Blunt/TA Ligase Master Mix (M0367, NEB). The ligation reaction was incubated for 10 min on room temperature. After 10 min 1 μl HP tether was added to the ligation and the reaction carefully mixed by inversion. After mixing, the reaction was incubated for another 10 min on room temperature. During incubation 50 μl Dynabeads MyOne Streptavidin C1 (Thermo Fisher) were washed twice in 100 μl Bead Binding Buffer on a DynaMag-2 Magnet (Thermo Fisher) and resuspended in 100 μl Bead Binding Buffer. The adapter-ligated, tether-bound DNA was mixed with 100 μl washed beads and incubated for 5 min on room temperature on an elliptical rotator. Afterwards the beads were pelleted on a DynaMag-2 Magnet and washed twice with 150 μl Bead Binding Buffer. The beads were resuspended in 25 μl Elution Buffer and incubated for 10 min at 37 °C. 1 μl of the eluate was quantified yielding 330.0 ng and 138.4 ng recovered DNA in total.

### MinION sequencing

The Conditioning Mix composed of 26.6 μl Fuel Mix, 550 μl 2× Running Buffer and 474 μl water was prepared on ice. Prior to loading a dry quality control step was performed to assess the number of available pores. After successful dry quality control of the flow cell 6 μl sequencing library was mixed with 75 μl 2× Running Buffer, 4 μl Fuel Mix and 65 μl water on ice to a total volume of 150 μl Sequencing Mix and the 48 h sequencing run was started. Before library loading the flow cell was conditioned twice for 10 min with 500 μl Conditioning Mix. Immediately after the two conditioning steps 150 μl Sequencing Mix were loaded on the flow cell. Briefly before 24 h run time another 150 μl freshly prepared Sequencing Mix were loaded on the R7.3 flow cell. The remaining library was stored in the fridge during this time period, the same library was sequenced on two flow cells.

### Raw Nanopore data processing

Base calling of raw sequencing data was performed using the Metrichor Agent 2.38.3 2D Basecalling for SQK-MAP006 workflow. FASTA and FASTQ format reads were extracted from downloaded HDF5 (fast5) format files with NanoOK [36]. Single sequence files were merged using the cat command on the UNIX command line. The quality of the merged FASTQ files was assessed using FASTQC-0.11.4 [37]. Read statistics was calculated with ABySS-1.5.1 [38], the modal read length was determined using a custom python script (available from: https://github.com/mgiolai/MinION_Ren-seq).

### Pre-assembly read filtering

Illumina and Oxford Nanopore adapter sequences in the MinION 2D pass and Illumina and SMRT-bell adapter sequences PacBio RoI were removed using cutadapt-1.8.1 [32] (Additional file 1: MinION/PacBio read adapter curaction and chimeric read removal). In the first step we removed 65 bases (the length of the Illumina adapter sequence) from both ends of a read using the cutadapt –u + 65 –u − 65 option. We further searched and removed adapter sequences in the MinION 2D pass reads and PacBio RoI by providing a FASTA file with the Illumina adapter, Illumina amplification primer, PacBio SMRT-bell or Oxford Nanopore adapter sequence and using the cutadapt –b option. We specified 20% adapter error rate for the MinION 2D pass reads and 5% adapter error rate for the PacBio RoI. Reads shorter than 150 bp were removed using cutadapt’s –m 150 flag. After trimming we used BLASR-1.3.1.142244 [33] to determine sequences which still contain adapters (Additional file 1: MinION/PacBio read adapter curaction and chimeric read removal). The reads highlighted by BLASR were removed from the sequence file using custom bash and python scripts (Additional file 1: MinION/PacBio read adapter curaction and chimeric read removal and github repository: https://github.com/mgiolai/MinION_Ren-seq). Read statistics of the filtered files were calculated using abyss-fac function of ABySS -1.5.1 [38].

### Determination of off-target rate

To determine how many reads and contigs contain at least one bait sequence we searched the read and contig sequences with the bait sequences using BLAST-2.2.29 blastn –task megablast –max_target_seqs 1 –max_hsps 1 [23] (Additional file 1: BLASTN to determine off-target capture rate). The BLAST results were filtered with a custom python script that only scores a hit if read and bait possess 80% sequence identity over 96 bp (available from: https://github.com/paajanen/Renseq).

### MinION 2D pass and PacBio reads of insert Canu assembly

The adapter filtered MinION 2D pass and PacBio RoI files were assembled using Canu-1.0 with default options by specifying a genome size of 9.0 Mb as reported by Witek et al. [11] using SMRT RenSeq and Geneious R8 [39] assembly. Adapter filtered MinION 2D pass files were Canu corrected and trimmed prior to assembly to increase the accuracy of the reads. The adapter filtered PacBio RoI were assembled without previous correction due to the high sequence accuracy – a strategy also followed up by Witek et al. for the Geneious assembly [11]. The MinION 2D pass data assembly was further polished with nanopolish-0.4.0 using default settings [25].

### HGAP assembly

As a further control to the proposed MinION assembly pipeline we performed a PacBio RoI data assembly based on a HGAP [26] pipeline which has been already reported to assemble NLR genes in *Solanum verrucosum* [12]. This pipeline is based on a modification of the standard SMRT-analysis 2.3.0 pipeline. The main steps different to the standard SMRT-analysis assembly pipeline are filtering the raw reads from Illumina adapters using BLASR [33] and whitelisting to filter out reads where adapter sequences are not removed. In this pipeline the edited bax.h5 files are fed into the SMRT-analysis HGAP3 protocol, with a parameter file that containing the whitelisting (available from: https://github.com/paajanen/Renseq).

### Read and contig quality control

As reference for evaluating sequencing read and assembly data we used a FASTA file containing 649 complete NLR genes of *S. americanum* accession 954,750,186 (working name SP2271) released by Witek et al. [11]. Lower accuracy reads (MinION 2D pass and PacBio Subreads) were mapped to this reference using bwa-0.7.13 [40, 41] with the –x ont2d flag (−k14 -W20 -r10 -A1 -B1 -O1 -E1 -L0). Higher accuracy reads (Canu corrected MinION 2D pass and PacBio RoI) and assembled contigs were mapped to the NLR gene reference using the –x pacbio flag (−k17 -W40 -r10 -A1 -B1 -O1 -E1 -L0). Mapping statistics was assessed using Qualimap-1.0 [42]. To determine the percentage of sequence identity and alignment length between single reads and full length NLR gene sequences we searched the NLR gene reference with the reads using BLAST-2.2.29 [23]. To assess if all full length NLR genes characterised by Witek et al. [11] are also present in the Canu and HGAP assemblies and to determine the percent identity, alignment length and alignment location of the full length NLR gene in the assembled contigs, we searched the assemblies with the full length NLR gene sequences using BLAST-2.2.29 blastn –task megablast –max_target_seqs 1 –max_hsps 1 [23]. Only BLAST results with an e-value of 0 were considered in the evaluation for each alignment. To filter the BLAST output file, we used a custom python script (Additional file 1: BLASTN to search NB-LRR database with assembled contigs and github repository: https://github.com/mgiolai/MinION_Ren-seq) creating a tabular output. We further hand-curated the dataset by removing false positive hits produced by the same subject sequence. The average values and standard deviation of the percent identity and alignment lengths in the obtained tables were calculated from this file using Microsoft Office Excel 2013.

### NLR protein motif analysis

NLR protein motif-encoding reads and contigs were determined using the NLR-Parser-1.0 [24] using default values.

### MUMer assembly comparison

We used the MUMer-3.23 [43] package to compare the assemblies. We aligned the Canu and HGAP assemblies to the Geneious reference assembly using NUCmer [27] using default settings except setting minmatch length to “–l 500”. Dot plots of NUCmer alignments were generated with mummerplot (Additional file 1: MUMer analysis to compare assemblies).

### NLR protein sequence prediction

We used AUGUSTUS-3.1 [29] with the options –uniqueGeneId = true –strand = both –genemodel = partial –gff3 = on –species = tomato to predict protein sequences on the assemblies and the 649 NLR gene reference. The predicted proteins were analysed with the NLR-Parser [24] by running the MAST [28] motif scan directly on the amino acid sequences. For comparison we searched the predicted proteins of the Canu, HGAP and Geneious assemblies with the predicted protein sequences of the 649 NLR gene reference using BLAST-2.2.29 [23] blastp –max_target_seqs 1 –max hsps 1. We handcurated the dataset by removing false positive hits being produced by the same subject sequence.

### Pilon MinION contig repair

As MinION error profiles are predominantly indels (which cause frameshifts) [15, 36] and Illumina errors are predominantly mismatches [44] -the error types are complementary- we tested effect of paired end Illumina sequences based correction. Illumina RenSeq described in [11] were trimmed for Illumina adapter sequences (setting 5% adapter error rate) and the quality threshold –q 20 using cutadapt-1.8.1 [32]. After trimming we converted the FASTQ files to the FASTA format and used BLASR-1.3.1.142244 [33] to identify sequences still containing adapters . The reads highlighted by BLASR were removed from the sequence file using a custom python script (Additional file 1: MinION read adapter curation and chimeric read removal and github repository: https://github.com/mgiolai/MinION_Ren-seq). We merged the produced FASTA files and mapped all reads to the nanopolished Canu MinION 2D assembly using bwa-0.7.13 [40, 41] without modified settings. The nanopolished Canu MinION assembly was repaired using Pilon-1.18 [30] with Pilon’s default settings and the flags –changes –fix all.

### Prediction of novel NLR genes

A reference database was constructed by manually removing the 37 fusion domain NLR genes from the 649 NLR gene reference. We used BLAST-2.2.29 [23] blastn –task megablast -max_target_seqs 1 -max_hsps 1 to search this reference dataset with the Canu corrected MinION reads and PacBio RoI. We extracted all the non-mapping reads using a custom python script (Additional file 1: Prediction of novel NLR genes and github repository: https://github.com/mgiolai/MinION_Ren-seq). We also analysed the BLAST results with MS Excel 2013. We removed all reads below the defined threshold of the percent identity minus two times the standard deviation of the percent identity. The remaining reads were extracted using a custom python script (available from: github repository: https://github.com/mgiolai/MinION_Ren-seq).

We pooled and assembled all non-mapping and low-quality mapping reads using the Canu –nanopore-corrected option and a genome size of 0.1 Mb. We saw few differences in assembly performance from varying the genome size while using the Canu -nanopore-corrected flag. We searched the 649 NLR gene reference database with the contigs using BLAST [23] blastn –task megablast -max_target_seqs 1 -max_hsps 1 and analysed the contigs by the NLR-Parser software [24].

## Additional file

Additional file 1 The amplification trace of the capture library and R7.3 Nanopore RenSeq flow cell yields. All commands used for data analysis (PDF 652 kb)

## Abbreviations

Bp: base-pair
ETI: Effector triggered immunity
HR: Hypersensitive response
Kbp: Kilobase-pair
Mbp: Megabase-pair
NLR: Nucleotide-binding leucine-rich repeat
ONT: Oxford Nanopore Technologies
PAMP: Pathogen associated molecular pattern
PTI: Pattern- or PAMP-triggered immunity
RenSeq: Resistance-gene enrichment sequencing
RoI: Read of Insert
SMRT: Single-molecule real-time
SR: Subread
ZMW: Zero-mode waveguide
