# Supplementary material for: Comparative analysis of targeted long read sequencing approaches for characterization of a plant’s immune receptor repertoire
Source: BMC Genomics. 2017 Jul 26;18:564. doi: 10.1186/s12864-017-3936-7 (PMC5530509; doi:10.1186/s12864-017-3936-7)
Supplement: Additional file 1 — The amplification trace of the capture library and R7.3 Nanopore RenSeq flow cell yields. All commands used for data analysis (PDF 652 kb) [file 12864_2017_3936_MOESM1_ESM.pdf]

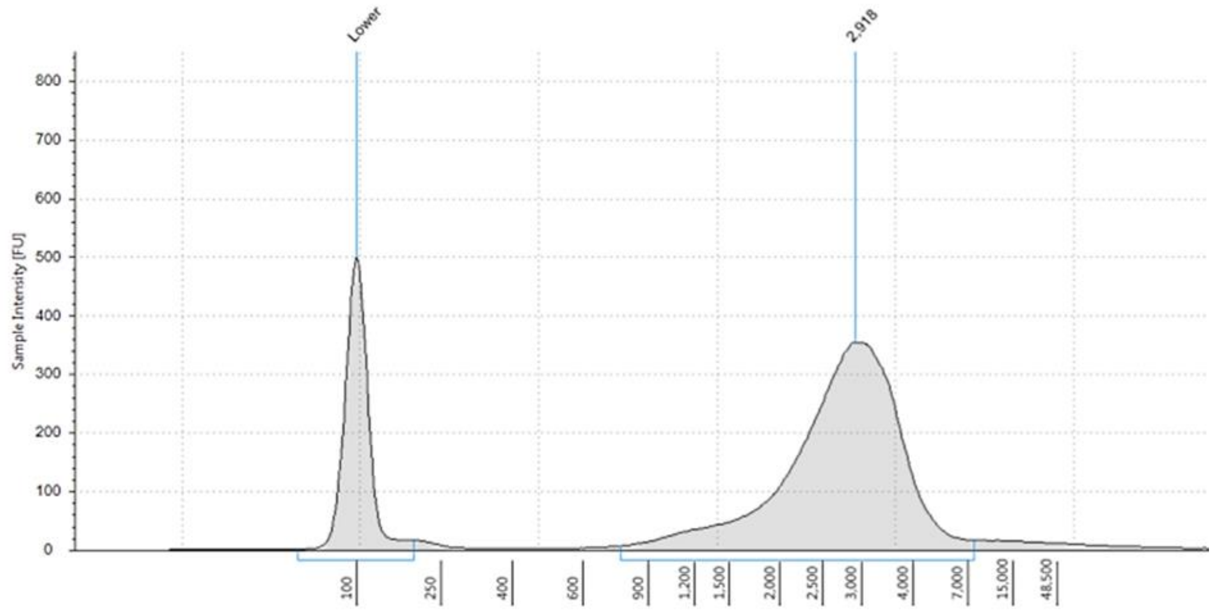

Figure S1 Kapa HiFi PCR amplification trace of the captured NLR genes sequences. The first peak at 300 bp (annotated with Lower) belongs to the loaded marker.

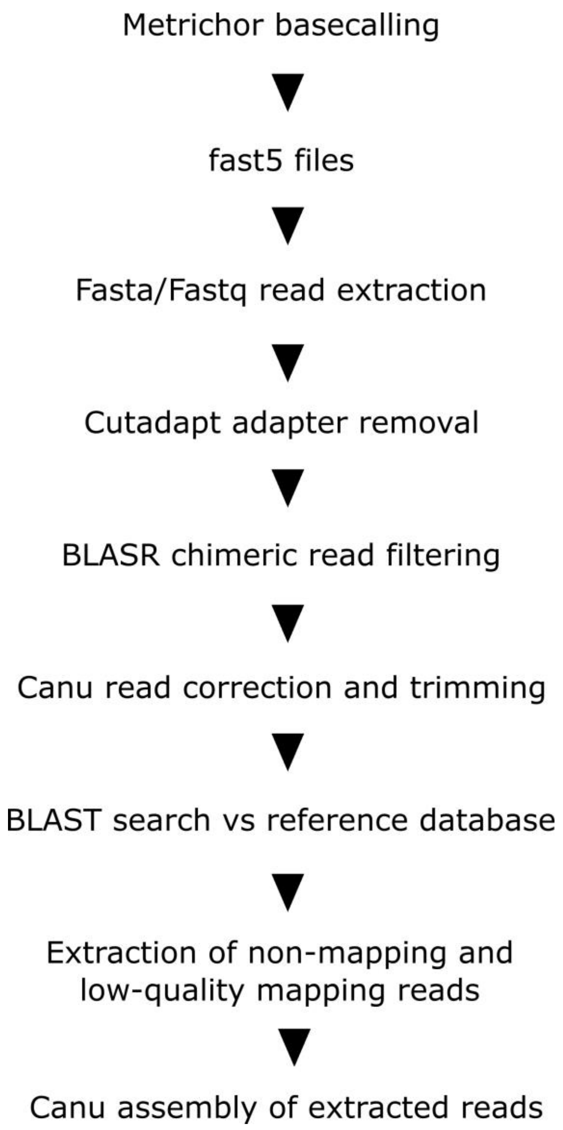

Figure S2 Proposed pipeline to predict novel NLR genes in comparison with a reference R-gene database: Reads are trimmed for adapters and corrected with Canu. The corrected reads are used to BLAST search a reference database with NLR genes. Reads that are not mapping to this dataset or mapping with low quality are extracted and assembled with Canu.

Table S1 MinION R7.3 flowcell performance: Obtained reads from four R7.3 flowcell sequencing runs (Mbp calculated using abyss-fac).

	Template Fail	Complement fail	2D fail	Template pass	Complement pass	2D pass
**Flow cell 1**	226.0	75.3	56.0	203.0	194.0	203.0
**Flow cell 2**	302.0	154.0	119.0	256.0	243.0	251.0
**Flow cell 3**	34.0	13.2	10.0	12.0	11.5	12.0
**Flow cell 4**	67.6	30.1	24.2	36.2	35.1	37.2
**Total**	629.6	272.6	209.2	507.2	483.6	503.2

<u>MinION read adapter curation and chimeric read removal</u>

(1) Cutadapt trimming

```
#!/bin/bash

cutadapt –u 65 –u -65 o {CUTADAPT FASTA OUTPUT FILE #1} {FASTA SEQUENCE INPUT FILE}

cutadapt -b file:{ADAPTER SEQUENCE FASTA FILE} -e 0.20 -m 150 -o {CUTADAPT FASTA OUTPUT FILE2} {CUTADAPT FASTA OUTPUT FILE #1}
```

(2) BLASR search for chimeric reads

```
#!/bin/bash

blasr {CUTADAPT FASTA OUTPUT FILE2} {ADAPTER SEQUENCE FASTA FILE} -m 1 -bestn 10 -out {BLASR OUTPUT FILE}.m4
```

(3) Filter reads using python (creates new fasta file without chimeric reads)

```
python MinION2D-filter.py {CUTADAPT FASTA OUTPUT FILE} {BLASR OUTPUT FILE}.m4 {FASTA OUTPUT FILE}
```

<u>PacBio read adapter curation and chimeric read removal</u>

(1) Cutadapt trimming

```
#!/bin/bash

cutadapt –u 65 –u -65 o {CUTADAPT FASTA OUTPUT FILE #1} {FASTA SEQUENCE INPUT FILE}

cutadapt -b file:{ADAPTER SEQUENCE FASTA FILE} -e 0.05 -m 150 -o {CUTADAPT FASTA OUTPUT FILE2} {CUTADAPT FASTA OUTPUT FILE #1}
```

(2) BLASR search for chimeric reads

```
#!/bin/bash

blasr {CUTADAPT FASTA OUTPUT FILE} {ADAPTER SEQUENCE FASTA FILE} -m 1 -bestn 10 -out  {BLASR OUTPUT FILE}.m4
```

(3) Filter reads using python (creates new fasta file without chimeric reads)

python PacBio-filter.py {CUTADAPT FASTA OUTPUT FILE} {BLASR OUTPUT FILE}.m4 {FASTA OUTPUT FILE}

MUMer analysis to compare assemblies

```
#!/bin/bash

comp={VARIABLE NAME}

srun nucmer -l 500 -p ${comp} {REFERENCE ASSEMBLY FILE} {QUERY ASSEMBLY FILE}

srun show-coords -THrcl ${comp}.delta > ${comp}.coords

if [ -s ${comp}.coords ]; then

        srun mummerplot --postscript -l --prefix ${comp} ${comp}.delta

        srun ps2pdf ${comp}.ps

fi
```

BLASTN to determine off-target capture rate

```
#!/bin/bash

makeblastdb -in {SEQUENCE FILE FOR DATABASE} -dbtype nucl -out {DATABASE}

blastn -task megablast -query {QUERY SEQUENCE FILE} -db {DATABASE} -num_threads 8 -
max_target_seqs 1 -max_hsps 1 -outfmt "6 qseqid sseqid pident length mismatch gapopen qstart
qend sstart send evalue bitscore qlen slen" -out output.blast.txt

python parse-blast-for-bait-hits.py output.blast.txt > {OUTPUT}.txt
```

BLASTN to search NB-LRR database with assembled contigs

```
#!/bin/bash
```

makeblastdb -in {SEQUENCE FILE FOR DATABASE} -dbtype nucl -out {DATABASE}

blastn -task megablast -query {QUERY SEQUENCE FILE} -db {DATABASE} -num_threads 8 -max_target_seqs 1 -max_hsps 1 -outfmt "6 qseqid sseqid pident length mismatch gapopen qstart qend sstart send evalue bitscore qlen slen" -out output.blast.txt

python blast-assembly-besthit-evaluation.py output.blast.txt > besthit-evaluation.txt

<u>Prediction of novel NLR genes</u>

(1) <u>BLASTN to NB-LRR reference</u>

#!/bin/bash

makeblastdb -in {FASTA FILE CONTAINING READS} -dbtype nucl -out {DATABASE}

blastn -task megablast -query {QUERY SEQUENCE FILE} -db {DATABASE} -num_threads 8 -max_target_seqs 1 -max_hsps 1 -outfmt "6 qseqid sseqid pident length mismatch gapopen qstart qend sstart send evalue bitscore qlen slen" -out output.blast.txt

(2) <u>Extract non-mapping reads</u>

python extract-nonmapping-reads.py {FASTA FILE CONTAINING READS} output.blast.txt > {FASTA OUTPUT FILE}

(3) <u>Extract badly mapping reads</u>

Remove all the entries in the BLAST output.blast.txt file manually that are not to be extracted.

python extract-badmapping-reads.py {FASTA FILE CONTAINING READS} {MANUALLY EDITED output.blast.txt} > {FASTA OUTPUT FILE}

(4) Canu assembly

<u>MinION:</u>

#!/bin/bash

canu –assemble –p {PREFIX} –d {DIRECTORY} genomeSize=0.10m –nanopore-corrected {FASTA OUTPUT FILE}

PacBio:

#!/bin/bash

canu –assemble –p {PREFIX} –d {DIRECTORY} genomeSize=0.10m –pacbio-corrected {FASTA FILE WITH FILTERED NON-MAPPING OR LOW QUALITY MAPPING READS}

(5) NLR-Parser

#!/bin/bash

java -jar Translate6Frame.jar -i {CANU ASSEMBLY FASTA FILE} -o {SIXFRAME FASTA OUTPUT FILE}

srun mast {MOTIF FILE} {SIXFRAME FASTA OUTPUT FILE}

java -jar NLR-Parser.jar -i mast.xml -o output.mast.txt

(6) NLR-Parser result python filtering

python NLRparser-sequence-extract.py output.mast.txt {CANU ASSEMBLY FASTA FILE} > {FASTA OUTPUT FILE}
